# Supplementary material for: Long-term consumption of hydrogen-rich water provides hepatoprotection by improving mitochondrial biology and quality control in chronically stressed mice
Source: PLoS One. 2025 Feb 14;20(2):e0317080. doi: 10.1371/journal.pone.0317080 (PMC11828380; doi:10.1371/journal.pone.0317080)
Supplement: S1 Raw images — (PDF) [file pone.0317080.s002.pdf]

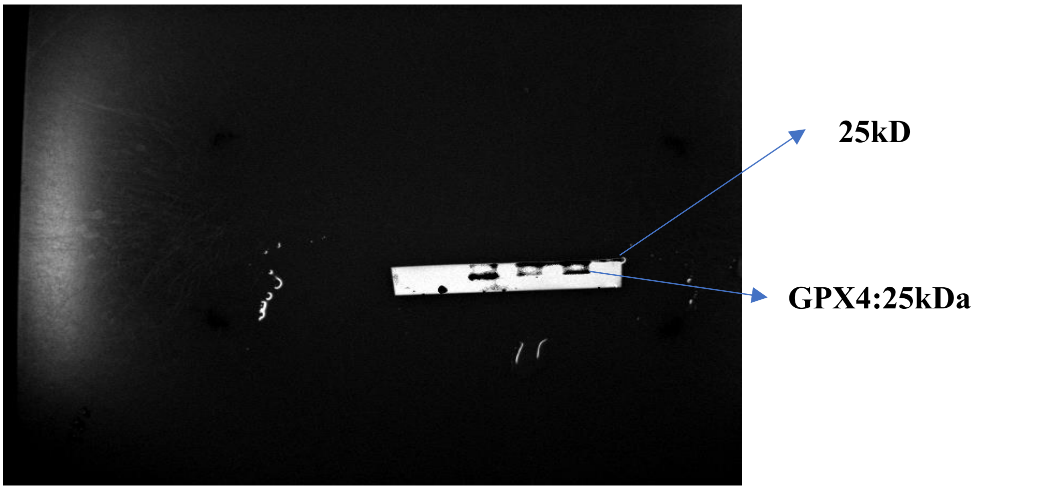

GPX4 (Figure 6)

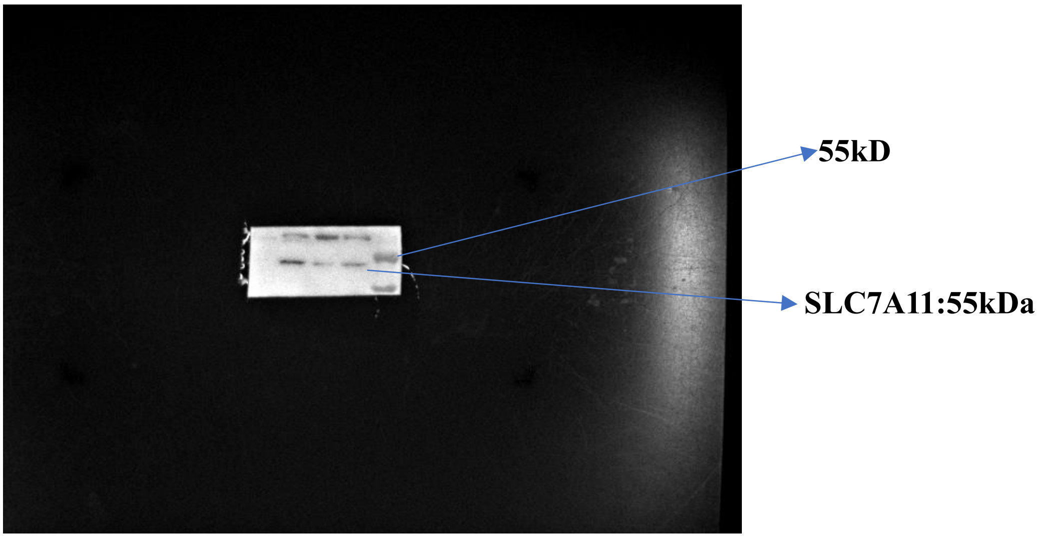

SLC7A11 (Figure 6)

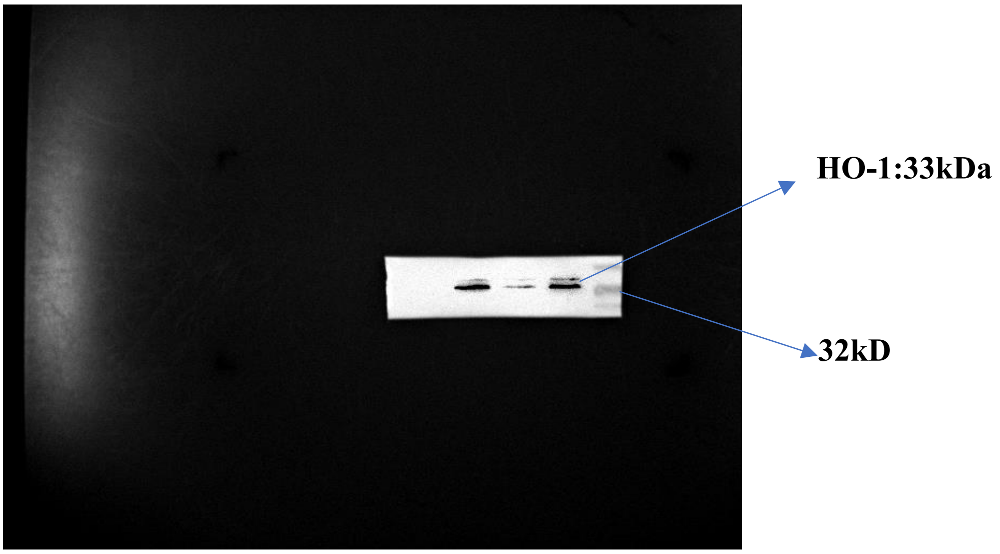

HO-1 (Figure 6)

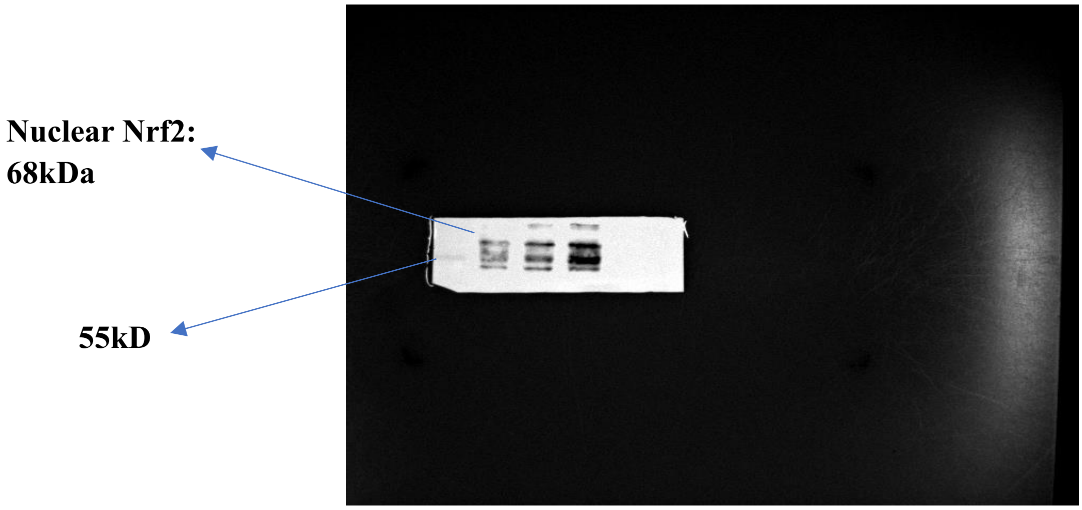

Nuclear Nrf2 (Figure 6)

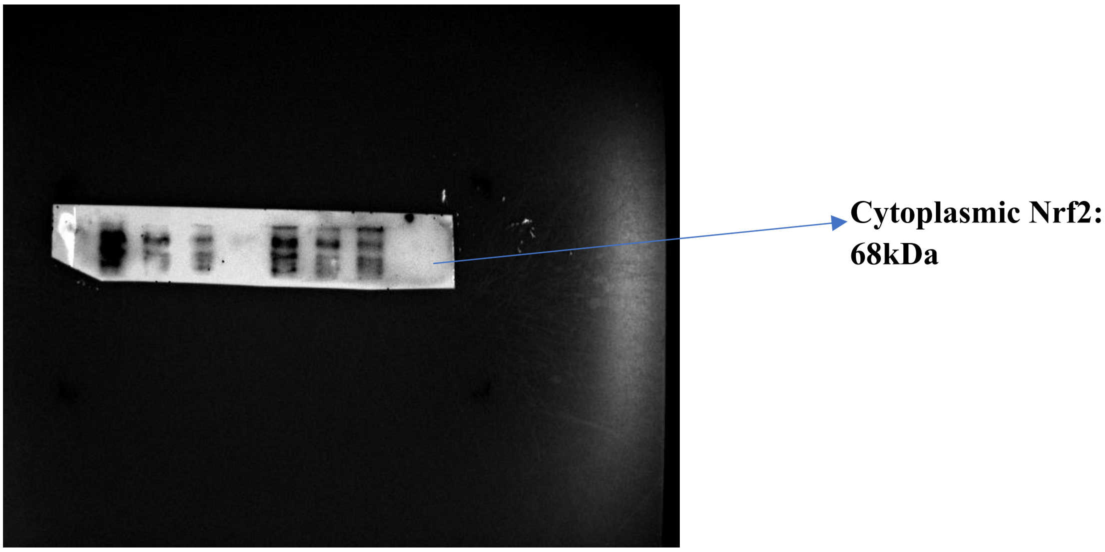

Cytoplasmic Nrf2 (Figure 6)

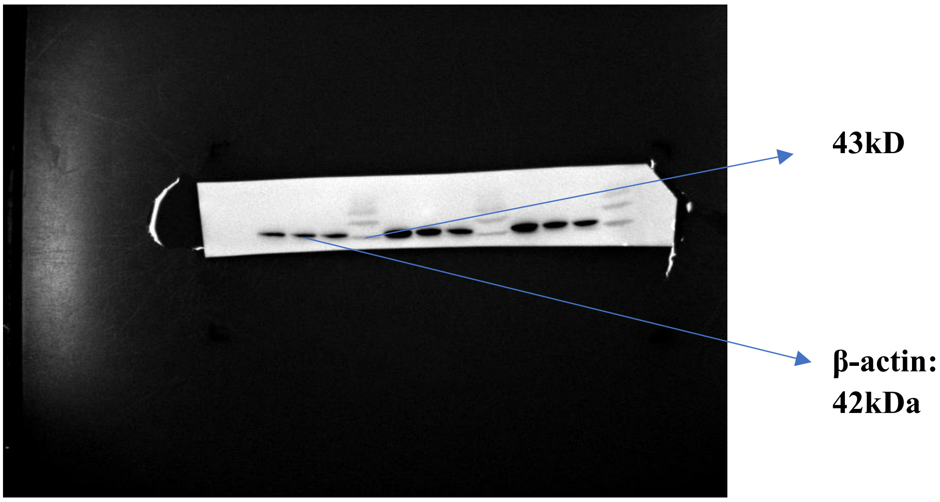

β-actin (Figure 6)

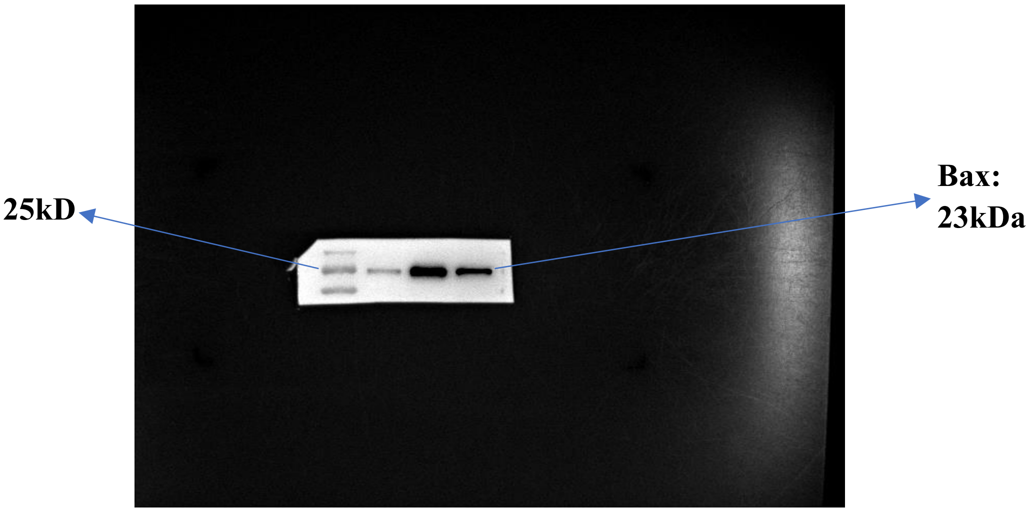

Bax (Figure 7)

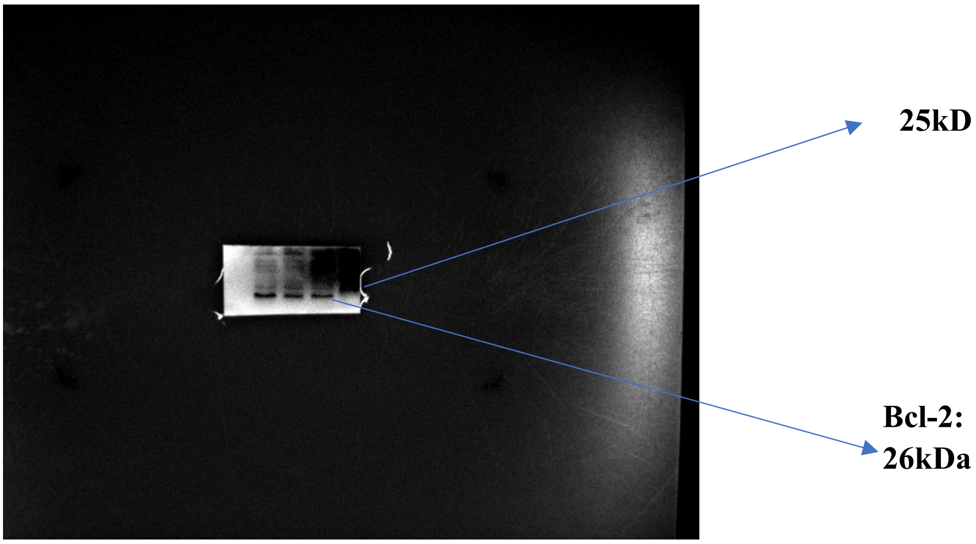

Bcl-2 (Figure 7)

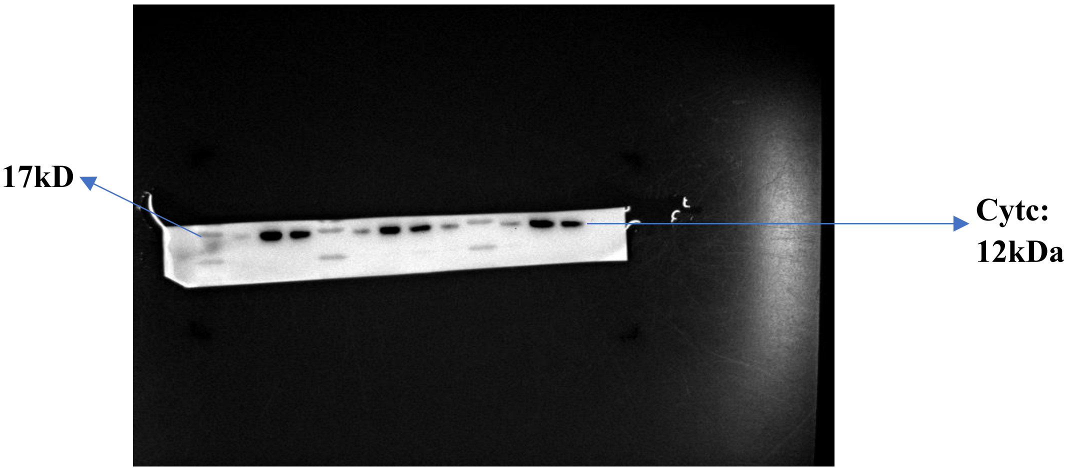

Cytc 9 (Figure 7)

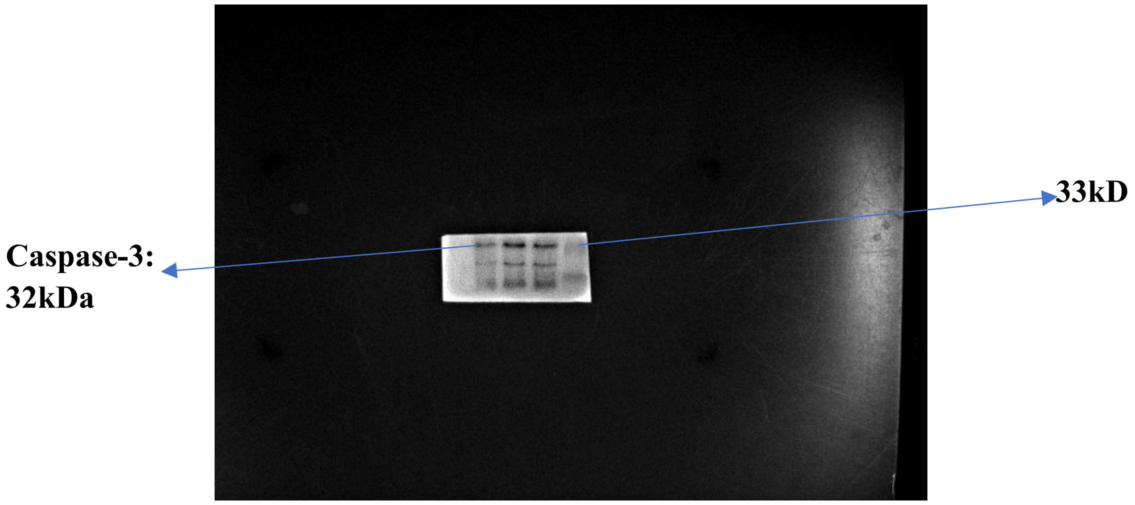

Caspase-3 (Figure 7)

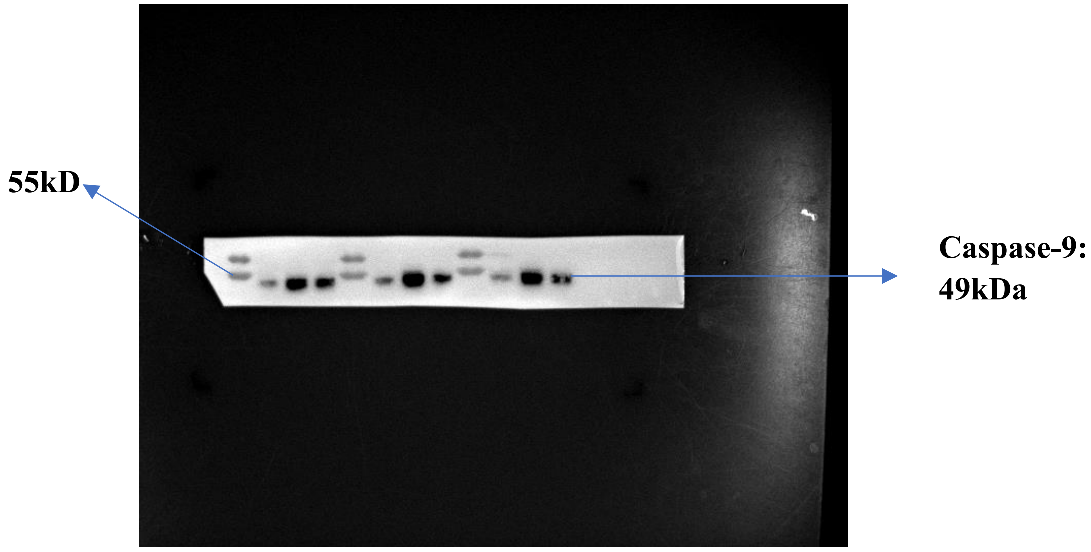

Caspase-9 (Figure 7)

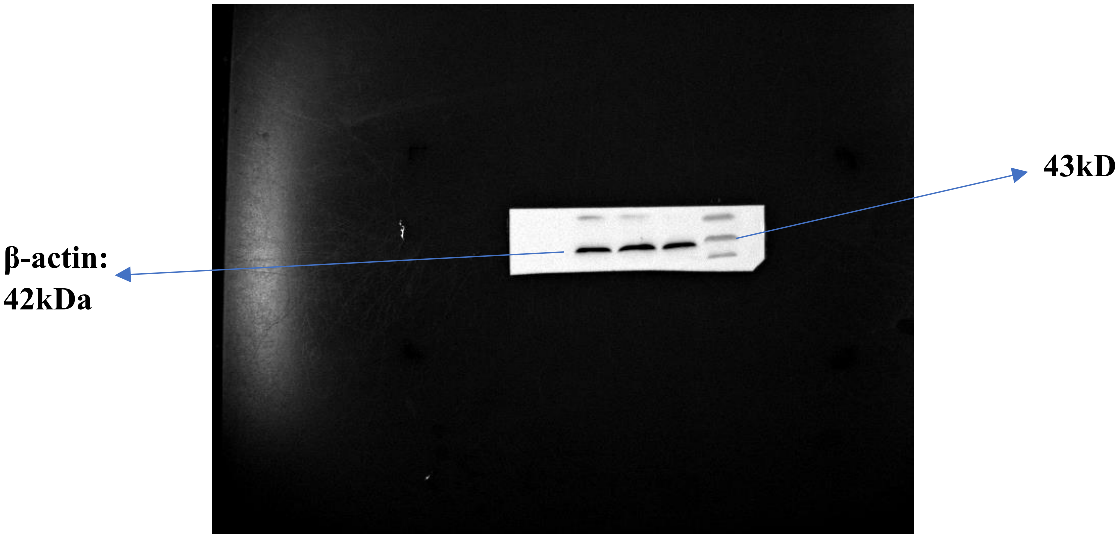

β-actin (Figure 7)

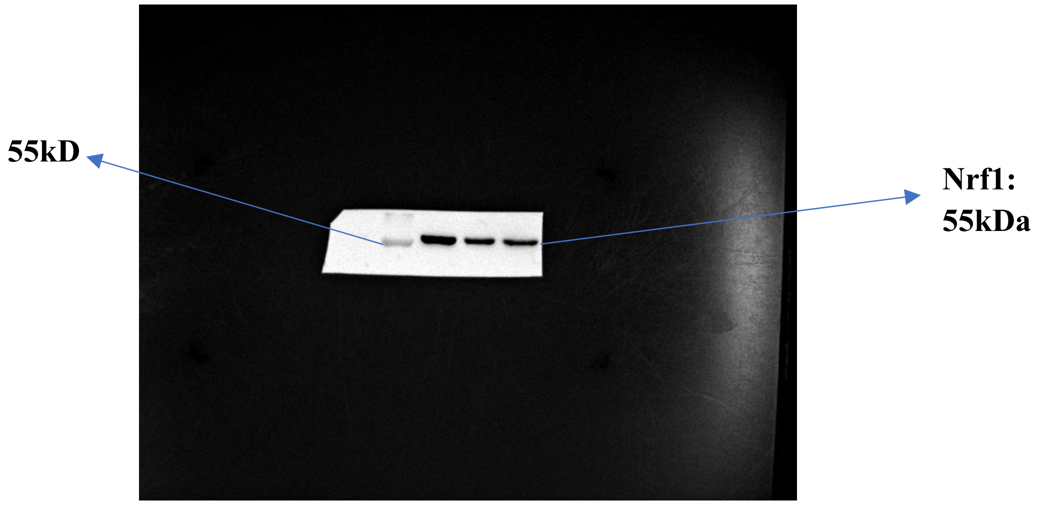

Nrf1 (Figure 8)

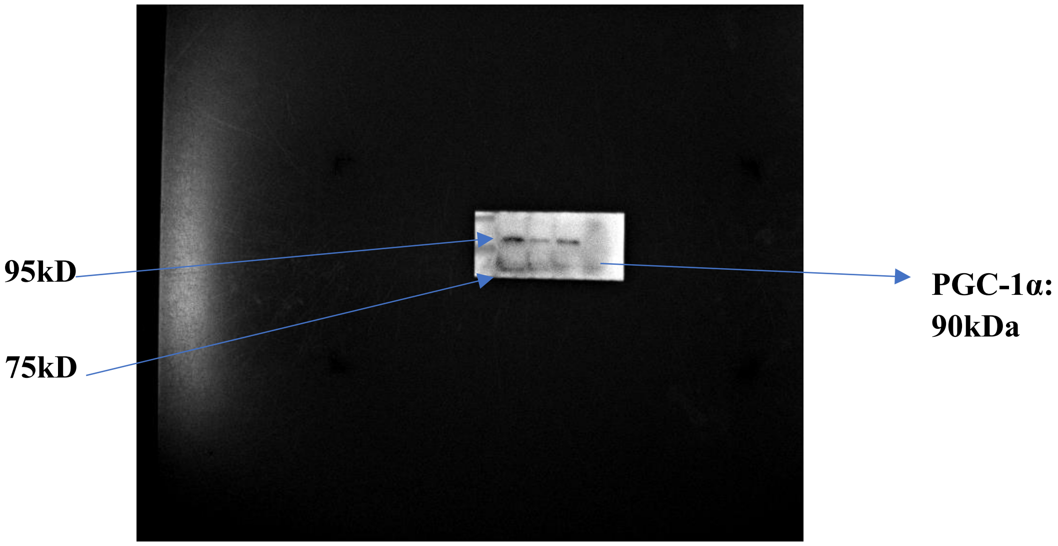

PGC-1α (Figure 8)

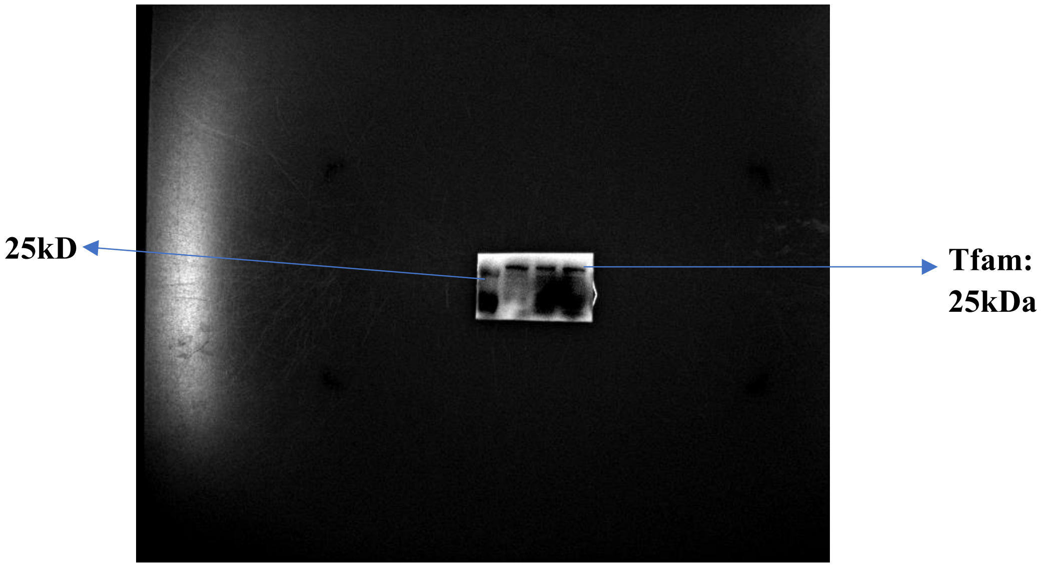

Tfam (Figure 8)

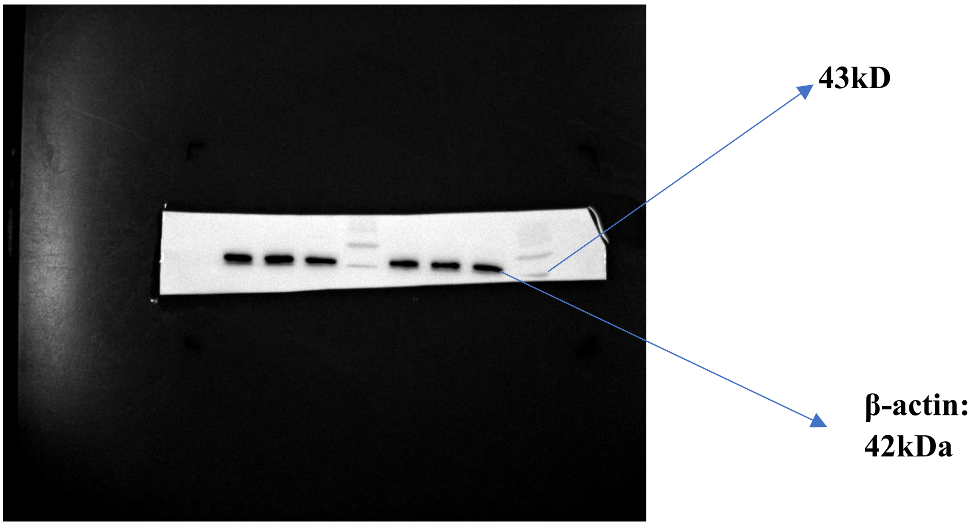

β-actin (Figure 8)

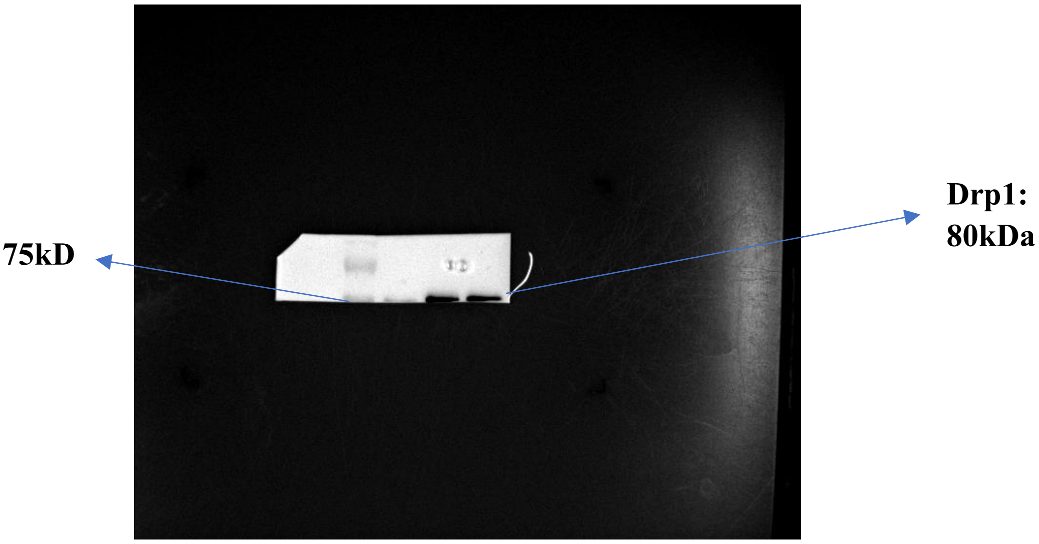

Drp1 (Figure 9)

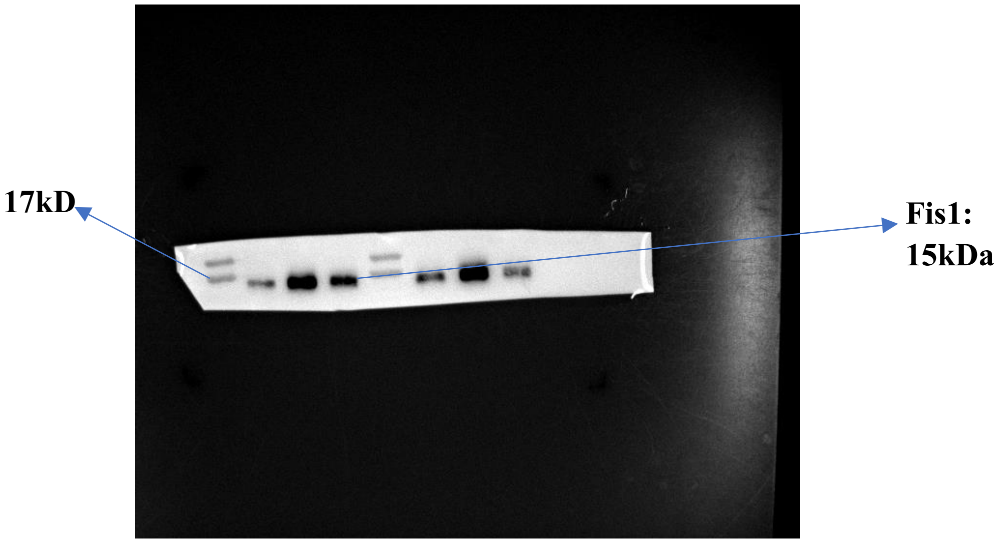

Fis1 (Figure 9)

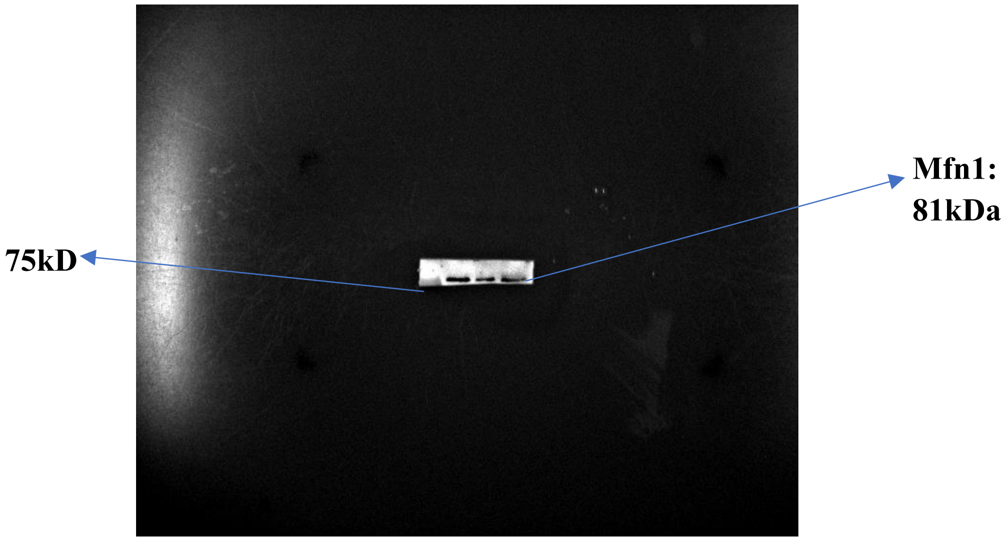

Mfn1 (Figure 9)

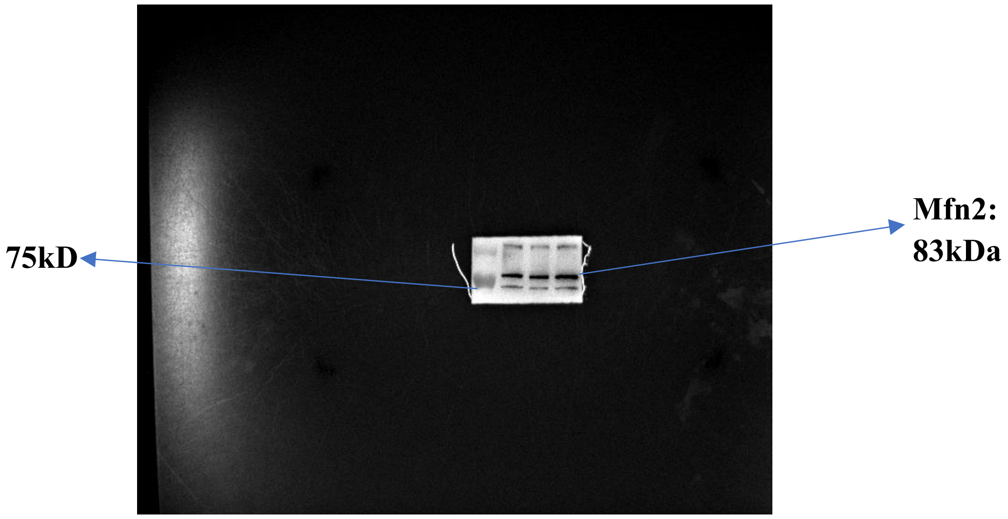

Mfn2 (Figure 9)

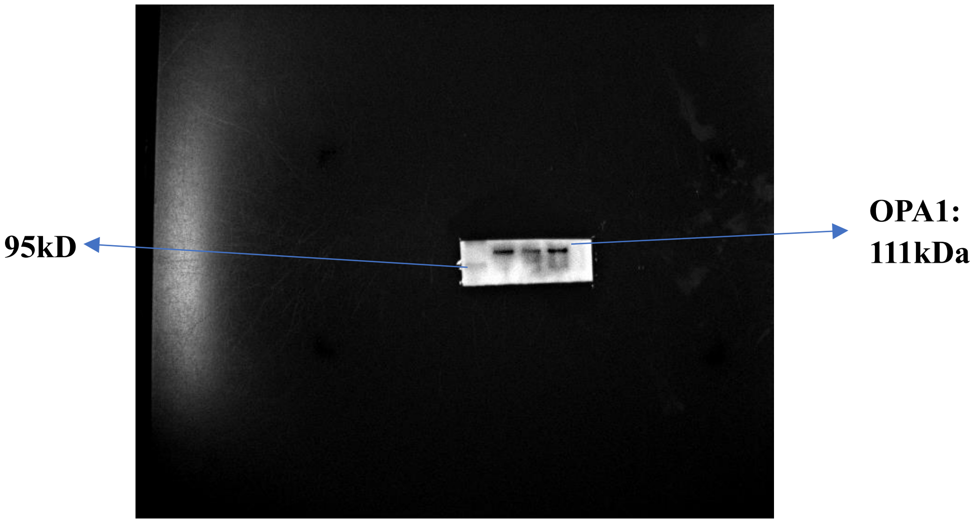

OPA1 (Figure 9)

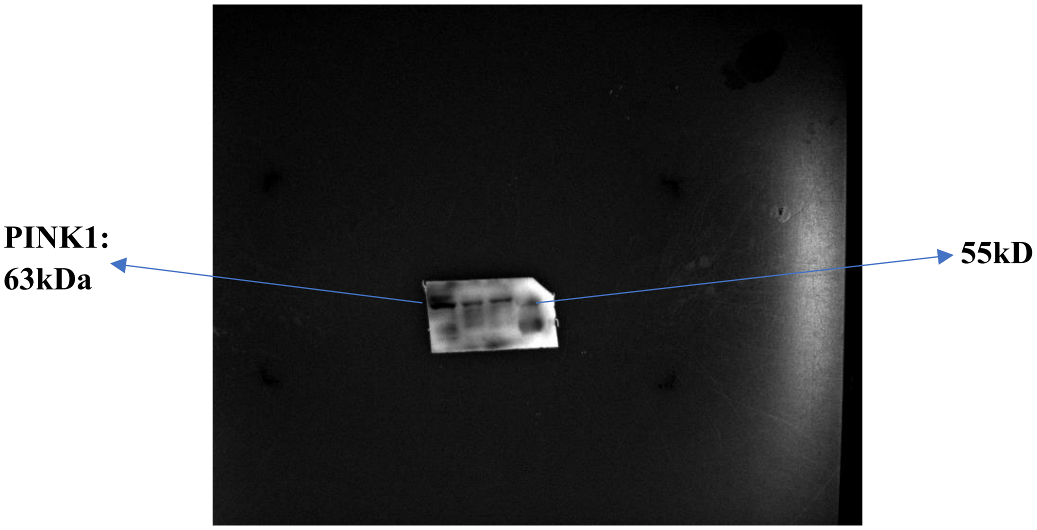

PINK1 (Figure 9)

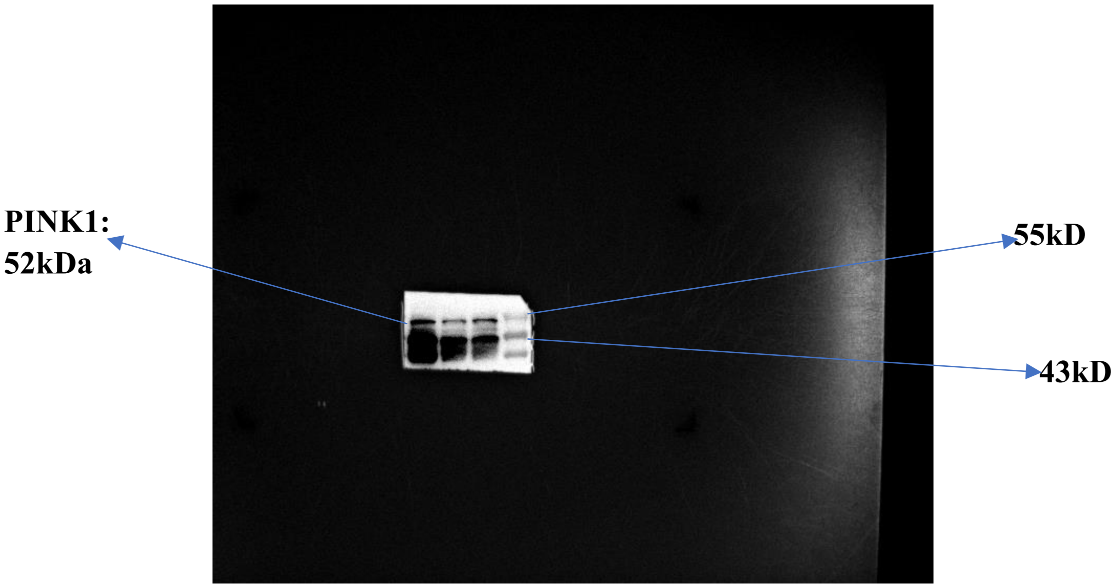

Parkin (Figure 9)

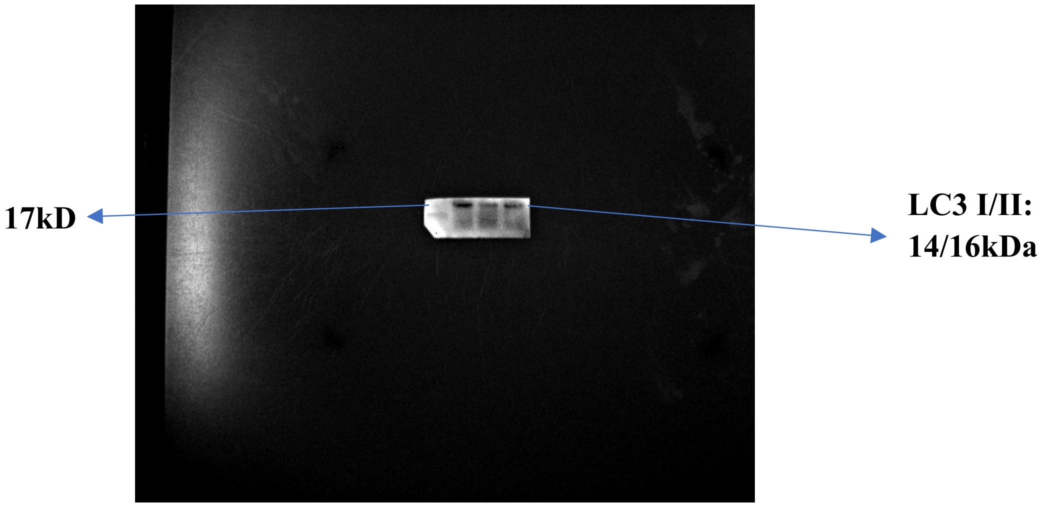

LC3 I/II (Figure 9)

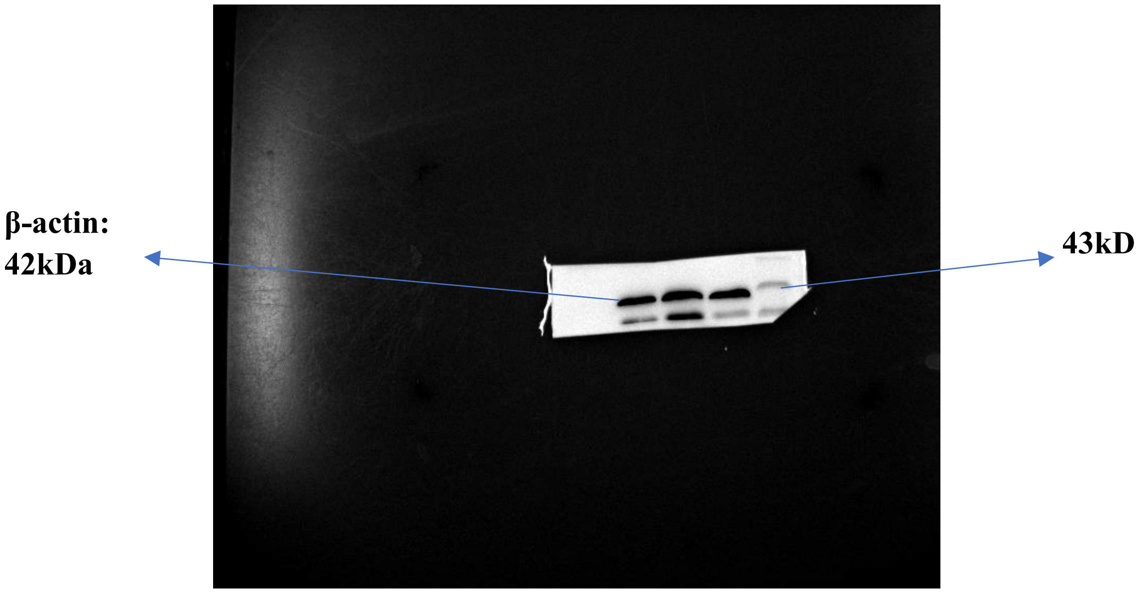

β-actin (Figure 9)

Supplyment gel

Figure 6

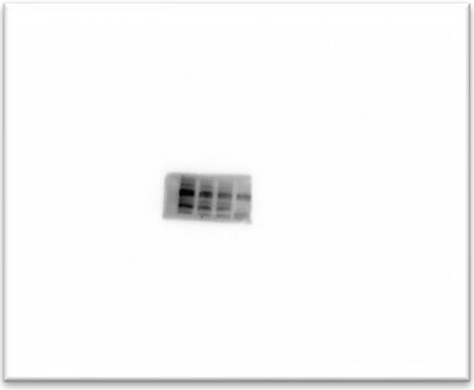

Supplyment cytoplasm Nrf2 (3)

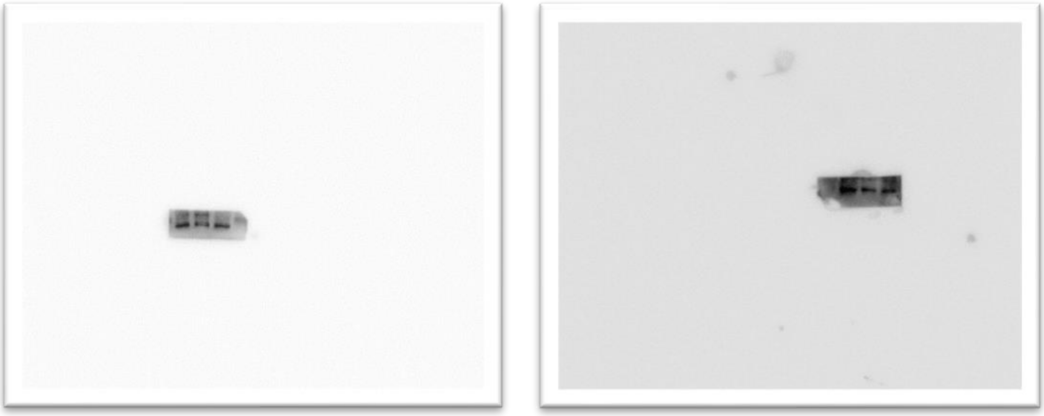

Supplyment GPX4 (2.3)

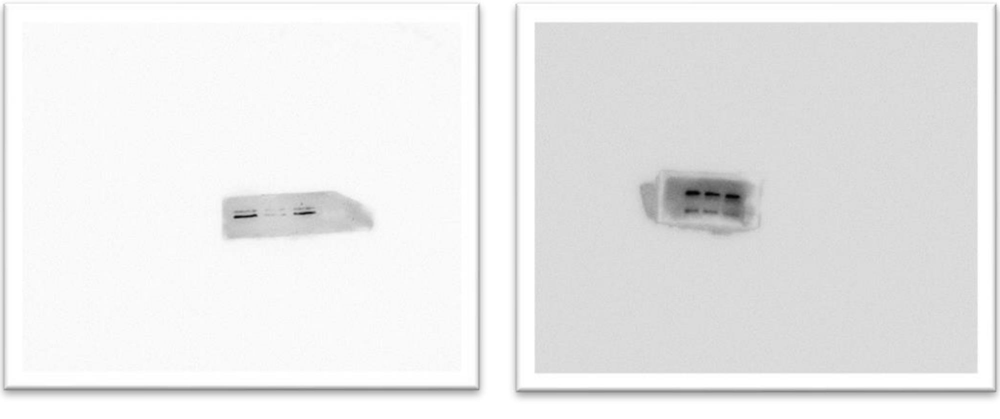

Supplyment HO-1 (2.3)

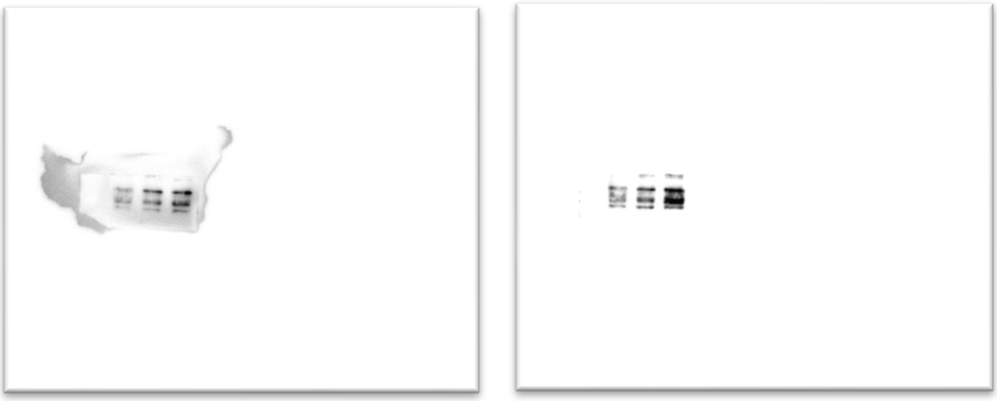

Supplyment Nuclear Nrf2 (2.3)

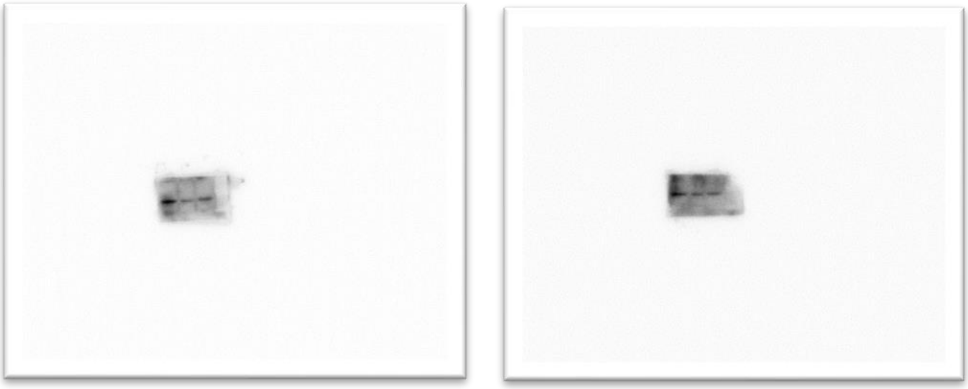

Supplyment SLC7A11 (2.3)

Figure 7

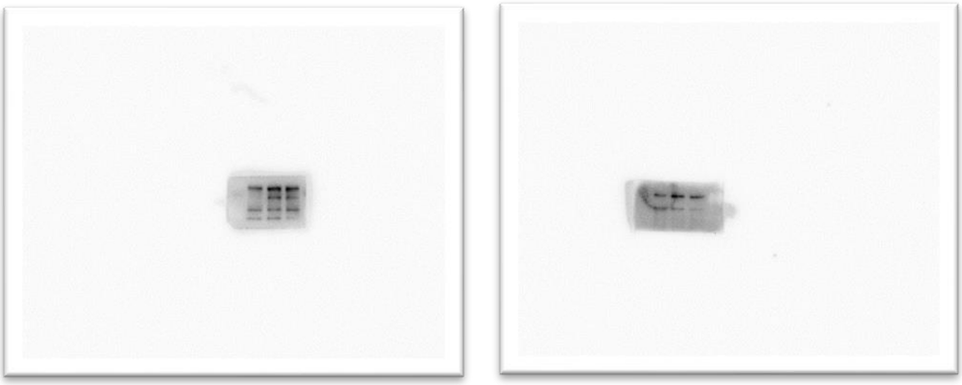

Supplyment Bax (2.3)

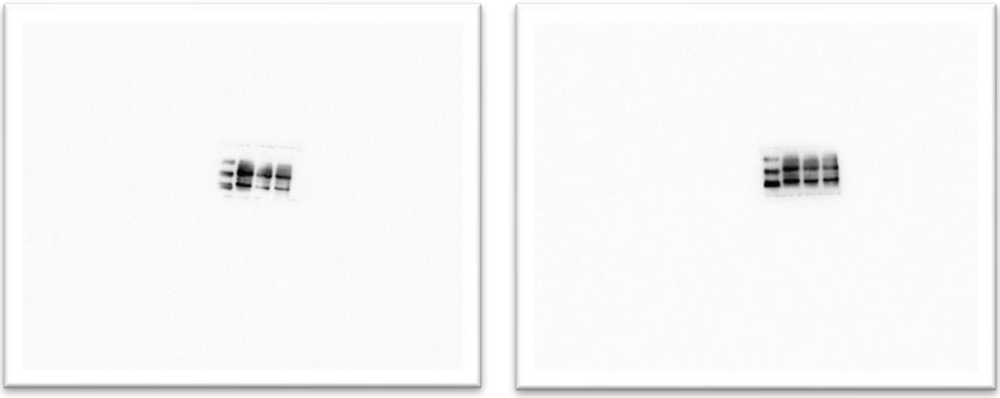

Supplyment Bcl-2 (2.3)

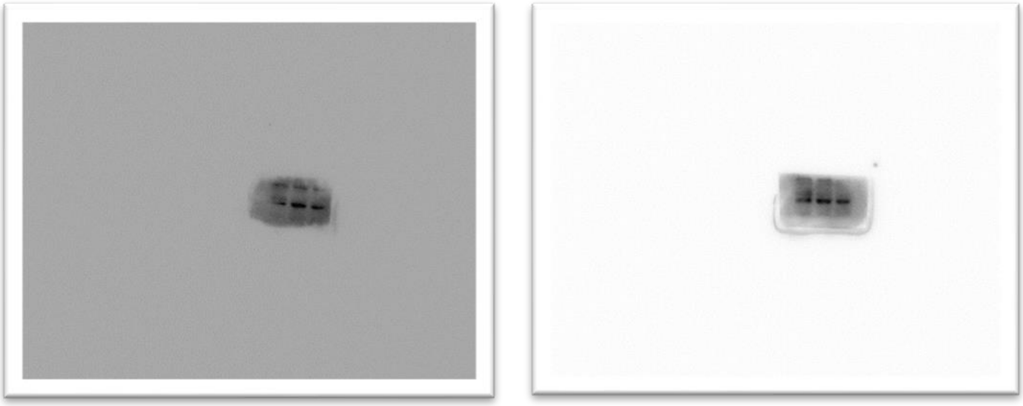

Supplyment Caspase-3 (2.3)

Figure 8

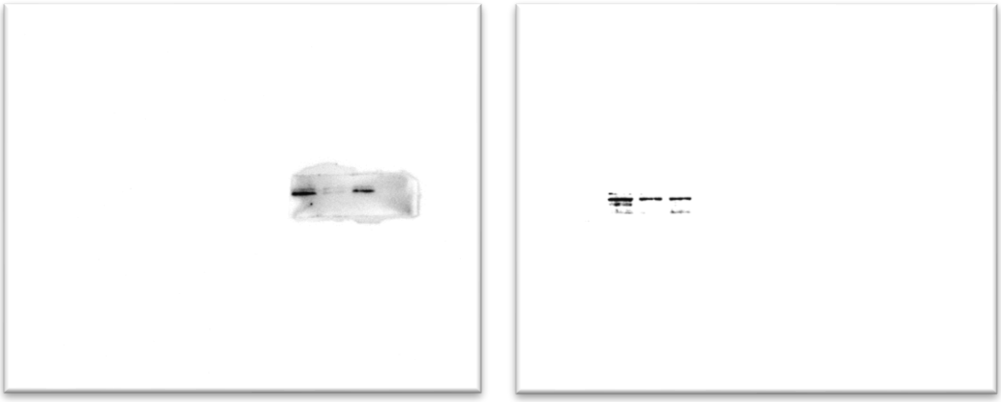

Supplyment Nrf1 (2.3)

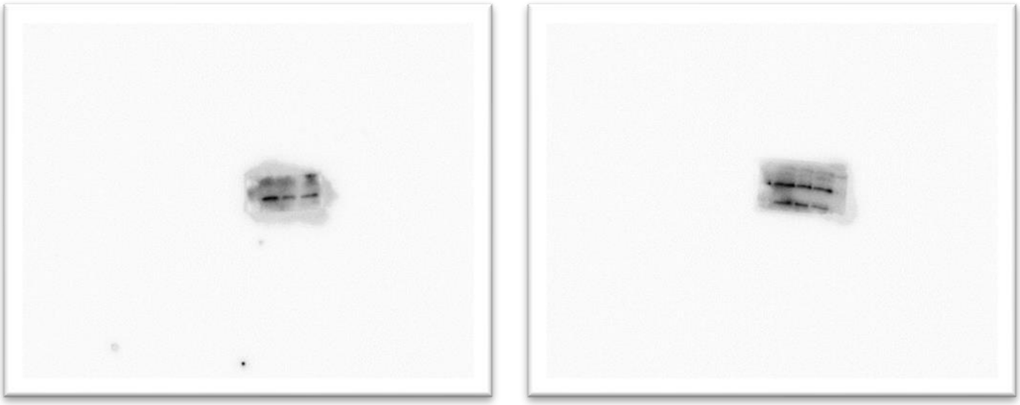

Supplyment PGC-1α (2.3)

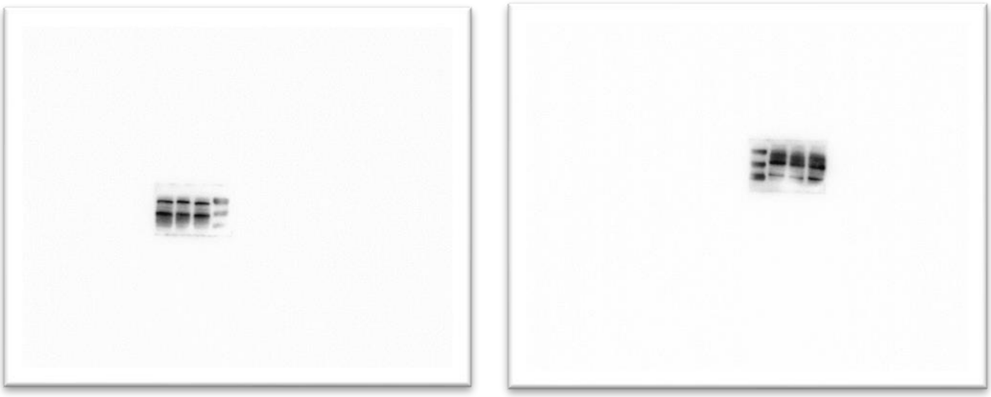

Supplyment Tfam (2.3)

Figure 9

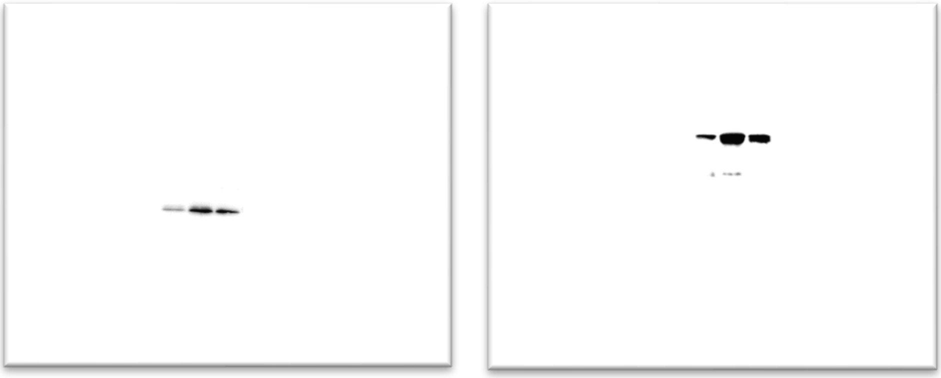

Supplyment Drp1 (2.3)

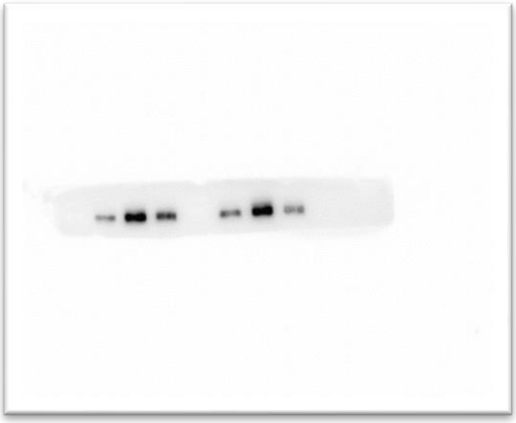

Supplyment Fis1 (1.2)

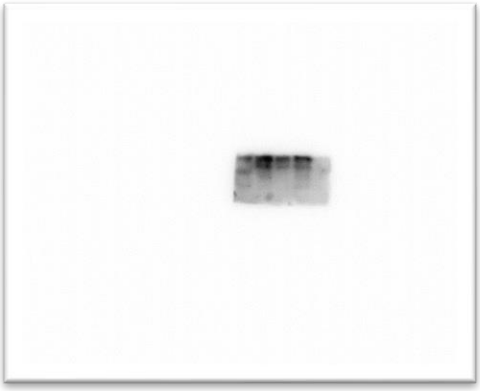

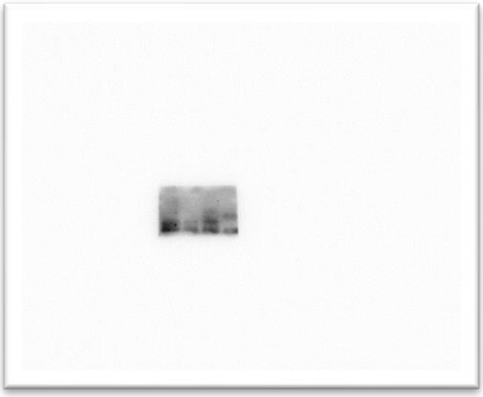

Supplyment LC3 (2.3)

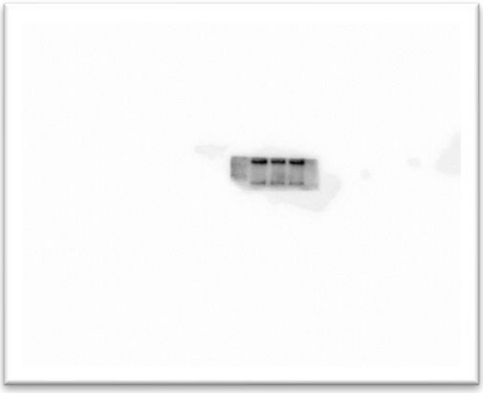

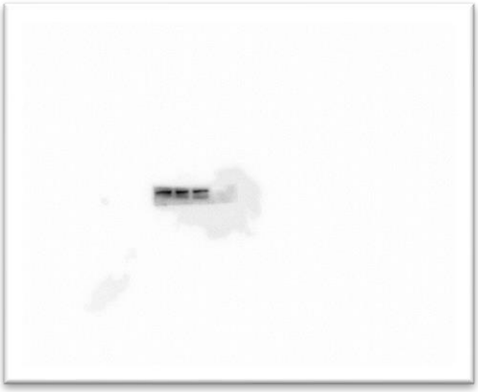

Supplyment Mfn1 (2.3)

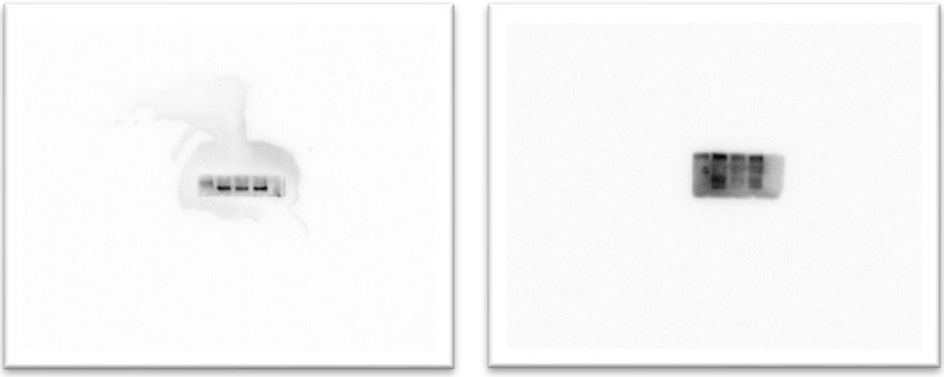

Supplyment Mfn2 (2.3)

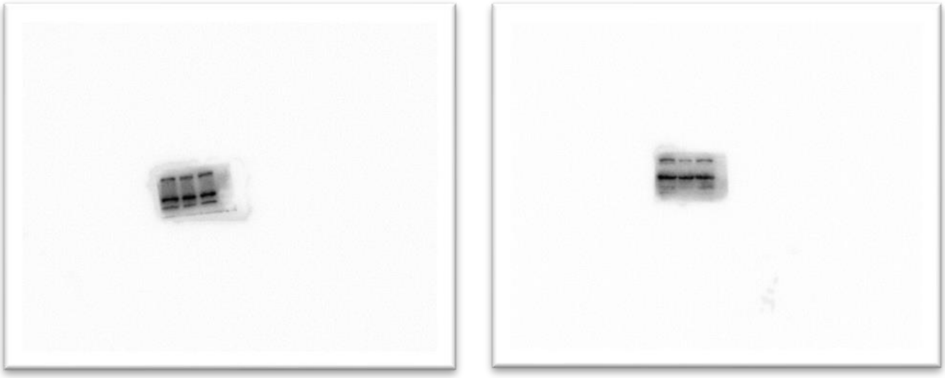

Supplyment OPA1 (2.3)

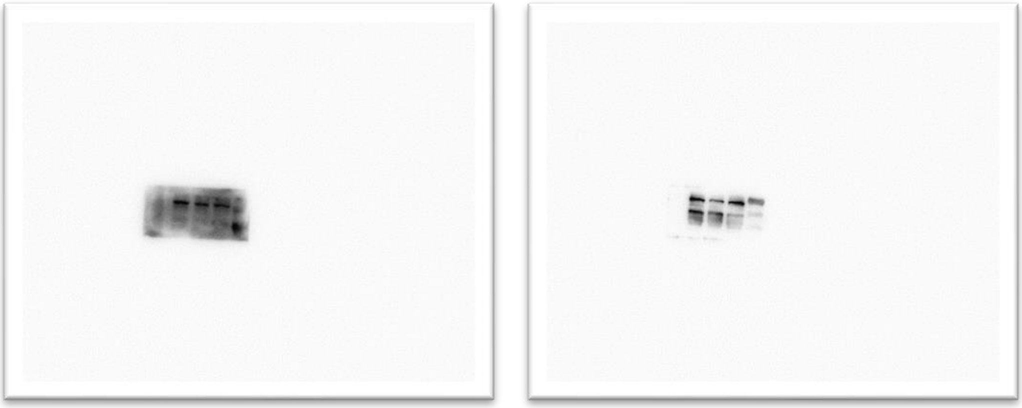

Supplyment Parkin (2.3)

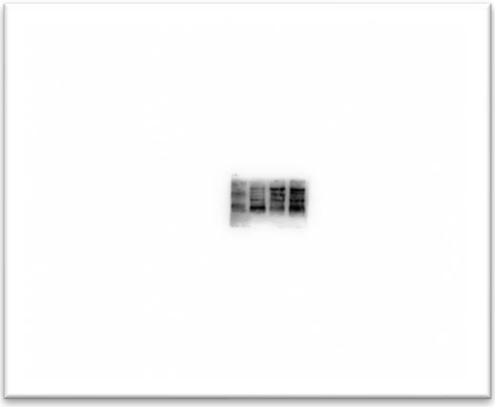

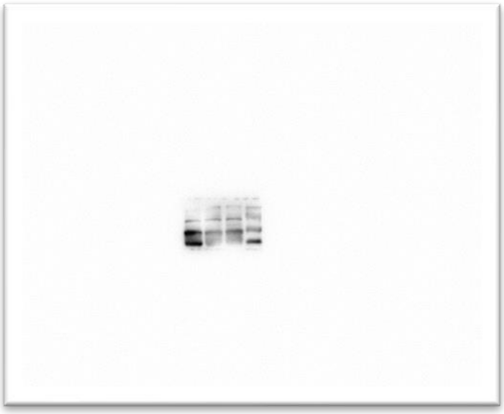

Supplyment Pink1 (2.3)

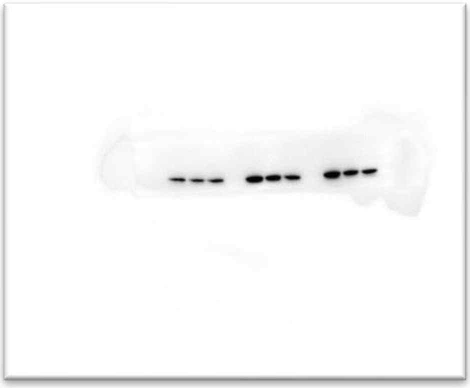

β-actin (1)

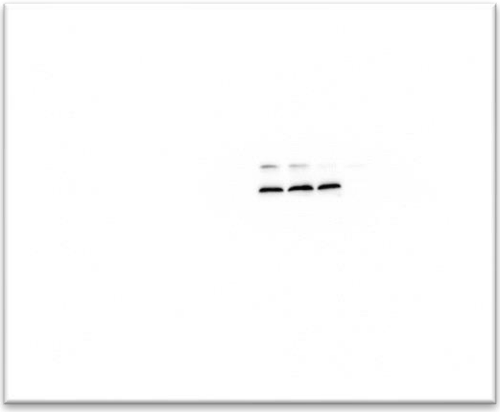

β-actin (2)

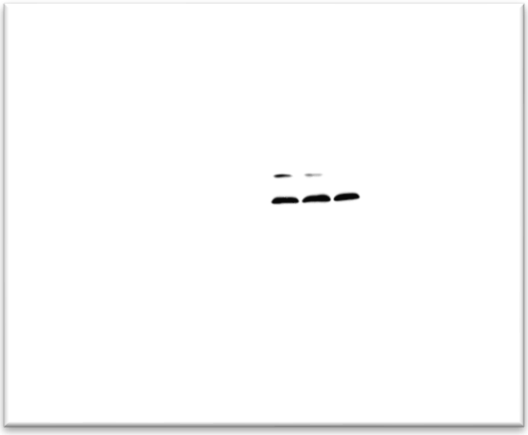

β-actin (3)

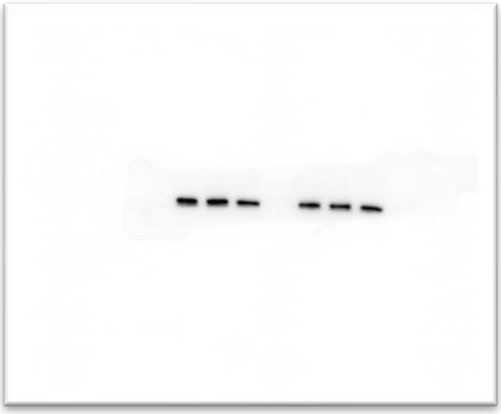

β-actin (4)
