# Supplementary material for: Long-term consumption of hydrogen-rich water provides hepatoprotection by improving mitochondrial biology and quality control in chronically stressed mice
Source: PLoS One. 2025 Feb 14;20(2):e0317080. doi: 10.1371/journal.pone.0317080 (PMC11828380; doi:10.1371/journal.pone.0317080)
Supplement: Attachment — Submitted filename: Response to reviewers.docx [file pone.0317080.s003.docx]

Response to reviewer 1:

Q1: Title: the title does not properly describe the content.
-I would like that the authors provide a title that highlight the potential antioxidant of Hydrogen rich water (HRW) on mitochondrial biogenesis and quality control.

Response: Thank you for your valuable feedback regarding the title of our paper. We appreciate your suggestion to highlight the potential antioxidant effects of HRW on mitochondrial biogenesis and quality control. We agree that the title should effectively convey the focus and significance of our research. We have revised the title (Long-term consumption of hydrogen-rich water provides hepatoprotection by improving mitochondrial biology and quality control in chronically stressed mice.) to better emphasize the antioxidant properties of HRW in relation to mitochondrial biogenesis and quality control. We believe this adjustment will enhance the clarity and relevance of our study.

Q2: Abstract: The abstract is sufficiently informative summary of the main aspect; however, it should be written in an intelligible fashion.

Response: We understand your concern regarding the clarity and intelligibility of the abstract. We have carefully revised it to ensure that the content is presented in a clear and accessible manner, making it easier for readers to understand the key findings and implications of our research.

Q3: Keywords: the keywords used may not allow a proper retrieval of the information on the internet.
Response: Thank you very much for your suggestion. We have updated the keywords of the article.

Q4: Abbreviation: the abbreviation used in the manuscript should be mentioned after keywords.
Response: Thank you very much for your suggestion. We have placed the abbreviation after the keyword.

Q5: Introduction: the first part of the manuscript is not an updated and interesting introduction to the subject. The authors should deeply enrich this section. According to the study the authors should give a summary of the recent studies on using Hydrogen-rich water and/or other close chemicals to alleviate oxidative stress damages in liver.

Response: Thank you for your insightful comments on the introduction section of our manuscript. We appreciate your suggestion to enrich this portion with more recent studies and information related to the use of Hydrogen-rich water and similar compounds in mitigating oxidative stress damages in the liver. We recognize the importance of providing an updated and engaging introduction that reflects the current state of research in this field. We have thoroughly reviewed recent literature and incorporate relevant studies that highlight the potential of Hydrogen-rich water and related substances in addressing oxidative stress-related liver damage.

Q6: The sub-title "Preparation of HRW" and "Establishment of CUMS model" must be included in the paragraph " Animals and experimental scheme" and Table 2-1 could be removed and write down how the CUMS was applied to the animals.

Response: Thank you very much for your feedback. We have made the relevant changes in the article as requested.

Q7: Line 94: KM is the abbreviation of what?
Response：KM mice is the abbreviation of Kunming mice.

Q8: Line 96-97: The authors must give more details here and provide the measurement followed to check the heath of the animals and that they were free from disease! "'The mice, all eight weeks old, were regularly checked for good health and did not have any underlying diseases before the start of the experiment."

Response: Thank you for your valuable feedback regarding the health assessment and disease screening procedures for the experimental animals in our study. The mice we used had undergone strict health checks at the biotech company before we purchased them, so we did not undergo too many checks during the week of adapting to the environment. We only conducted basic tests such as mental state, appetite, hair quality, and fecal condition.

Q9: Line 149: Elisa sub-title should be changed to Corticosterone determination and more details should be provided of how authors used the Elisa kit.

Response: Thank you very much for your suggestion. We have made the relevant changes in the article as requested.

Q10: Line 154: is it an expression level of Corticosterone?
Response: Yes, Cort is the abbreviation for corticosterone.

Q11: Material and methods for 4-Hydroxynoenal and MDA must be under the same Sub-title (Liver lipid peroxidation measurement).

Response: Thank you very much for your suggestion. We have made the relevant changes in the article as requested. Now this part is in Method 2.6 (Liver lipid peroxidation detection).

Q12: Line 156: Test kit_ the title of this paragraph must be changed.
Line 158 and 159: ALT and AST can be under the name of sub-title: biomarkers of liver toxicity.
Line 159 and 160: SOD, CAT and GSH as well can be in a different sub-title: Liver enzymatic antioxidant activities.

Response: Thank you very much for your suggestion. We have made the relevant changes in the article as requested. Now this part is in Method 2.4 and Method 2.5 (Biomarkers of liver toxicity; Liver enzymatic antioxidant activities).

Q13: The authors should provide a brief description of how each parameter was extracted and measured.

Response: Thank you for your feedback. I have supplemented the parameter measurement method in the Method.

Q14: Line 164: The authors can move the histological examination to the end and provide more details; the type of the optical microscope used and how the severity of stress was examined. Any scores on a scale were used?

Response: Thank you very much for your suggestion. We have supplemented the model of the microscope in the article. We use typical pathological changes in HE slices to determine the degree of stress, such as the arrangement of liver cords, cellular vacuolization, and bleeding in the liver. We did not use any scores as the severity of liver structural damage can be clearly observed in HE slices.

Q15: For the statistical analysis, I would like if the authors used the letters for significance.
Principal component analysis (PCA) is recommended to be applied in this study.

Response: Thank you for your suggestion regarding the application of Principal Component Analysis (PCA) in our study. We appreciate your interest in exploring alternative analytical approaches to enhance the interpretation of our data.
While PCA is indeed a powerful method for dimensionality reduction and pattern recognition, we chose to employ one-way ANOVA for several reasons specific to the objectives and design of our study.
Firstly, our primary aim was to examine the effects of different experimental conditions on specific outcome variables, such as antioxidant levels or mitochondrial function markers. One-way ANOVA allows us to assess the significance of differences among multiple groups and identify potential treatment effects.
Secondly, PCA is commonly used for exploratory data analysis and visualization, particularly when dealing with high-dimensional datasets. However, in our study, the variables under investigation were relatively well-defined and directly related to our research questions, making one-way ANOVA a more suitable choice for hypothesis testing and inference.
That being said, we acknowledge the value of PCA in identifying underlying patterns and relationships within complex datasets. In future studies, we will consider incorporating PCA as a complementary analytical tool to further explore the multivariate structure of our data.

Q15: Number all tables sequentially (Table 1, Table 2, etc.), likewise for figures (Figure 1, Figure 2, etc.).
In the title of each figure, authors should mention the number of biological replicates ± standard deviation. The title should be presented in an intelligible fashion.
-The quality of figures and histological section of liver must be improved.

Response: Thank you very much for your feedback. We have made the changes according to the relevant requirements.

Q16: The discussion part needs to be improved; it is missing the discussions with previous research in the same context (Example: 'Hydrogen-rich water reduces inflammatory responses and prevents apoptosis of peripheral blood cells in healthy adults: a randomized, double-blind, controlled trial, The Effects of 24-Week, High-Concentration Hydrogen-Rich Water on Body Composition, Blood Lipid Profiles and Inflammation Biomarkers in Men and Women with Metabolic Syndrome: A Randomized Controlled Trial, etc.).

Response: Thank you for your valuable feedback regarding the discussion section of our manuscript. We appreciate your insight into the need for a more comprehensive discussion, including discussions with previous research in the same context.
We understand the importance of contextualizing our findings within the broader body of literature and engaging in meaningful discussions with relevant previous research. In response to your suggestion, we have enhanced the discussion section by thoroughly integrating relevant studies and comparing our results with those reported in the existing literature.

Q17: The conclusion as well must be presented in an intelligible fashion.
Response: Thank you for your feedback regarding the conclusion section of our manuscript. We appreciate your emphasis on the need for clarity and intelligibility in presenting the conclusion. we have revised the conclusion section to ensure that it effectively communicates the main takeaways from our research in a manner that is easily understandable to our readers.

Q18: The discussion should not be repetitive for the results section.
Response: Thank you for your valuable feedback regarding the discussion section of our manuscript. We carefully reviewed and revised the discussion to ensure that it complements the results without repeating them verbatim.

Response to reviewer 2:

Q1: In this CUMS model, transaminases are elevated significantly in the model group. Is there any previous report which shows massive liver damage in CUMS models?
The reasons why the authors adopted this model for drinking hydrogen-rich water should also be explained.

Response: Thank you for your insightful questions regarding our choice of the Chronic Unpredictable Mild Stress (CUMS) model and the rationale behind studying the effects of hydrogen-rich water in this context.
Regarding previous reports demonstrating liver damage in CUMS models, while extensive literature exists on the behavioral and neurobiological effects of CUMS, reports specifically focusing on massive liver damage in CUMS models are relatively limited. However, there are studies suggesting that chronic stress can lead to hepatic dysfunction and alterations in liver enzymes, albeit the extent of liver damage may vary depending on the experimental conditions and duration of stress exposure. We will thoroughly review existing literature to provide a comprehensive discussion on the potential impact of CUMS on liver function and its relevance to our study. I have listed several relevant literature on CUMS induced liver injury.
(1) *Shuxie-1 Decoction Alleviated CUMS -Induced Liver Injury via IL-6/JAK2/STAT3 Signaling (DOI: 10.3389/fphar.2022.848355).* (2) *Dose-Effect/Toxicity of Bupleuri Radix on Chronic Unpredictable Mild Stress and Normal Rats Based on Liver Metabolomics (DOI: 10.3389/fphar.2021.627451).*
As for the rationale behind adopting the chronic stress model for studying the effects of hydrogen-rich water, chronic stress is known to trigger oxidative stress and inflammation, which are implicated in the pathophysiology of various stress-related disorders, including liver dysfunction. Hydrogen-rich water has gained attention for its potential antioxidant properties, which could counteract oxidative stress and mitigate its detrimental effects on liver function.

Q2: Hydrogen is known to selectively attenuate hydroxyl radicals and peroxynitrite (Nat Med. 2007 Jun;13(6):688-94. doi: 10.1038/nm1577). The antioxidant activity of hydrogen in this study appears to have a wide range of antioxidant activity, including SOD, CAT and GSH. The difference in the effects of hydrogen from previous reports should be discussed.

Response: Thank you for highlighting the comprehensive antioxidant activity of hydrogen. While Ohsawa et al.'s study provides valuable insights into the antioxidant properties of hydrogen, it's essential to recognize that the biological effects of hydrogen molecules on SOD/CAT/GSH.
*(1) The effect of hydrogen-rich water on letrozole-induced polycystic ovary syndrome in rats (DOI: 10.1016/j.rbmo.2023.103332).
(2) Hydrogen-rich water ameliorates rat placental stress induced by water restriction (DOI: 10.4103/2045-9912.241064).
(3) Local Treatment of Hydrogen-Rich Saline Promotes Wound Healing In Vivo by Inhibiting Oxidative Stress via Nrf-2/HO-1 Pathway(DOI: 10.1155/2022/2949824)*
In our study, we observed [insert findings related to hydrogen's effects on liver damage and antioxidant enzyme activity]. These results are consistent with the notion that hydrogen exerts potent antioxidant effects, which may help mitigate oxidative stress and preserve liver function in the context of chronic stress exposure.

Q3: The authors have shown that drinking hydrogen-rich water alleviates behavioral changes in response to chronic stress, but the mechanism underlying this has not been addressed. Many of the histological results in this paper indicate changes in ROS in the liver, but are there any previous reports that link this to behavioral changes? Or does the behavior-altering effect of hydrogen-rich water directly affect nerves? Suggestions for mechanisms of behavioral changes should also be mentioned.

Response: Thank you for your insightful comments regarding the mechanism underlying the behavioral changes observed in response to chronic stress and the potential link between liver ROS changes and behavioral alterations in our study.
We appreciate your acknowledgment of the focus of our experiment, which primarily aims to investigate the protective mechanism of hydrogen-rich water on liver damage induced by chronic stress. While behavioral changes are indeed a part of the validation data for establishing a chronic stress mouse model, we understand the importance of addressing the broader implications of our findings, including their impact on behavior.
While the precise mechanisms through which hydrogen-rich water influences behavior warrant further investigation, our study provides valuable insights into the potential therapeutic effects of hydrogen-rich water in mitigating the adverse effects of chronic stress on both liver and behavioral health.
Thank you for highlighting these important aspects, and we appreciate the opportunity to further explore the mechanistic underpinnings of our research findings.

Q4: What does Figure 3-7G mean? In the figure it is written as TUNEL staining, but in the figure legend it is written as Nrf2 immunofluorescent. Please describe correctly. Moreover, I can not see any fluorescent signal in the picture. Clearer figures are necessary.

Response：Thank you very much for your correction. I apologize for my basic mistake. I have changed the NRF2 in the legend to TUNEL and greatly improved the quality of the fluorescent images for your better viewing.
