# Supplementary material for: Long-term consumption of hydrogen-rich water provides hepatoprotection by improving mitochondrial biology and quality control in chronically stressed mice
Source: PLoS One. 2025 Feb 14;20(2):e0317080. doi: 10.1371/journal.pone.0317080 (PMC11828380; doi:10.1371/journal.pone.0317080)
Supplement: Attachment — Submitted filename: Response to reviewers.docx [file pone.0317080.s004.docx]

**Response to reviewers:**

Reviewer 1: Thank you for the update on the manuscript revision. While there has been notable progress in improving the Material and Methods section, the manuscript, particularly the Discussion and Conclusion sections, still require significant attention and improvement. Ensuring a thorough interpretation of the study's findings and accurate conclusions drawn from them is paramount, to enhance the manuscript's overall quality and impact.
-The manuscript requires significant improvement in adhering to standard English conventions. It's imperative to rectify this to uphold the manuscript's clarity, accuracy, and overall quality.
-Addressing these issues will ensure that the manuscript meets the necessary standards for publication.

Response: Thank you for your feedback on the revised manuscript. We appreciate your thorough evaluation and constructive comments.
We acknowledge your observations regarding the Discussion and Conclusion sections and recognize the importance of ensuring a thorough interpretation of the study's findings. We have reexamined these parts to provide a more comprehensive and accurate analysis, aligning the conclusions more closely with the study's results to enhance the manuscript's overall quality and impact.
Furthermore, we understand the significance of adhering to standard English conventions for clarity, accuracy, and overall quality. We have made efforts to solve this problem to ensure that the manuscript meets the necessary standards for publication.
Thank you once again for your valuable feedback. We are committed to making the required revisions to improve the manuscript in accordance with your suggestions.

Reviewer 2: The results of the present study are clinically important.
The authors did revised manuscript according to the Reviewer the request, appropriately.

Response: Thank you for acknowledging the clinical importance of the study's results. We greatly appreciate your recognition of the revisions made in response to the reviewer's feedback. We have strived to incorporate the suggestions appropriately to enhance the manuscript's quality and clarity.
If there are any further areas you believe require attention or if you have any additional feedback, please do not hesitate to let us know. We are committed to ensuring that the manuscript meets the necessary standards for publication.
Thank you for your continued support and guidance throughout this process.

Reviewer 3: The authors have done an excellent job in addressing the research questions regarding the hepatoprotective properties of hydrogen-rich water (HRW) in mice subjected to chronic stress. Their methods were well-detailed, allowing for a clear understanding of how the study was conducted. The results presented compelling evidence of HRW's beneficial effects on liver health, including histological improvements, normalization of liver function indicators, and modulation of various biochemical markers associated with oxidative stress, ferroptosis, and apoptosis.
However, while the content of the manuscript is comprehensive and scientifically sound, there is room for improvement in the flow of the presentation. The transition between sections could be smoother to enhance the coherence and readability of the paper. For instance, integrating a brief overview of the methods at the beginning of the results section could help readers better understand the context of the findings. Additionally, organizing the results in a more structured manner, perhaps grouping them based on the specific mechanisms or pathways examined, could improve clarity and facilitate the interpretation of the data. Please have a look at this article to discuss specific mechanism (https://www.mdpi.com/1422-0067/25/2/973).
Furthermore, the discussion section could benefit from a more nuanced exploration of the implications of the findings and their significance in the broader context of liver health research. While the conclusions succinctly summarize the key findings of the study, elaborating on the potential clinical relevance of HRW as a hepatoprotective intervention and discussing its limitations and future directions would provide a more comprehensive understanding of the research implications.
In summary, while the authors have made significant contributions to the understanding of HRW's hepatoprotective effects, refining the content flow and enhancing the depth of discussion would further strengthen the manuscript's impact and readability.

Q1: Can you please cite a few studies for this?
Moreover, numerous studies have reported that drinking hydrogen-rich water can increase hydrogen concentration in the liver, allowing hydrogen molecules to exert their effects within the liver.

Q2: Please rewrite the conclusion in 4-5 sentences without numbering them.

Response: Thank you for providing detailed feedback on our manuscript regarding the hepatoprotective properties of HRW in mice subjected to chronic stress. We appreciate your recognition of the thoroughness of our methods and the compelling evidence presented in the results section.
We agree with your assessment regarding the flow of the presentation and acknowledge the importance of enhancing coherence and readability. Integrating a brief overview of the methods at the beginning of the results section and organizing the results in a more structured manner, possibly grouping them based on specific mechanisms or pathways examined, are excellent suggestions. We have carefully reviewed the suggested article and conducted group discussions from a mechanistic perspective.
Additionally, we acknowledge the need for a more nuanced exploration of the implications of our findings in the discussion section. We have already discussed the limitations and future research directions of this study in the last paragraph, in order to provide deeper insights into the protective effects of hydrogen rich water on the liver.
In summary, we are grateful for your feedback and will work diligently to refine the content flow and enhance the depth of discussion to further strengthen the manuscript's impact and readability. Your guidance is invaluable in this process, and we are committed to addressing these suggestions to improve the overall quality of the manuscript.
Thank you once again for your thorough evaluation and constructive feedback.

Reply to Q1: We have cited relevant research articles at the end of this sentence.

Reply to Q2: We have removed the numbers and consolidated them into one paragraph.
