# Supplementary material for: Long-term consumption of hydrogen-rich water provides hepatoprotection by improving mitochondrial biology and quality control in chronically stressed mice
Source: PLoS One. 2025 Feb 14;20(2):e0317080. doi: 10.1371/journal.pone.0317080 (PMC11828380; doi:10.1371/journal.pone.0317080)
Supplement: Attachment — Submitted filename: Response to Reviewers.docx [file pone.0317080.s005.docx]

Reviewer #1:

Comment 1：The method outlined in the abstract is overly verbose.

Response:

Thank you to the reviewer for reviewing our work and providing valuable feedback. Regarding the issue you raised about the excessive length of the methods section in the abstract, we have streamlined the content multiple times while retaining the most essential information. However, due to the inclusion of multiple measurement indicators in this study, in order to ensure accurate communication of research methods, we have to provide a detailed description of the main method steps in the abstract.

We understand that the abstract needs to be concise, but at the same time, we also hope to provide sufficient information in this section so that readers can have a comprehensive understanding of the research design and main measurement indicators. Therefore, while minimizing non essential content, we chose to retain key methodological information related to the research.

We believe that this information is crucial for understanding the rigor of the research and the validity of the results, but we are also willing to optimize the expression as much as possible in further revisions to present the methodology section in a more concise and clear manner.
Thank you again for your feedback. We look forward to your further guidance and suggestions.

Comment 2: Ensure that the titles of tables and graphs are concise and descriptive, avoiding narrative language (e.i Table 1 As shown in the table, the type, brand, item number and dilution ratio of the first antibody factor used in the WB test)

Response：

Thank you to the reviewer for reviewing and providing suggestions for our work. Based on your feedback, we have simplified the titles of the tables and charts. After receiving your feedback, we specifically checked the title of "Table 1" and simplified it to "Table 1 Antibody Description”， To ensure that the title is both concise and descriptive, avoiding narrative language.

We believe that this modification can better meet your requirements and make the table content more intuitive and clear.

Thank you again for your valuable feedback. We look forward to your further guidance

Comment 3: Certain sections of the discussion reiterate the results unnecessarily. A discussion should primarily focus on comparing your findings with existing research. Please revise the discussion section to incorporate more references to other studies, rather than merely narrating the results.

Response:

Thank you to the reviewer for their careful review and feedback on our discussion section. Based on your suggestion, we have comprehensively rewritten the discussion section, with a focus on comparing the findings of this study with existing research. During the revision process, we removed redundant descriptions of the results to ensure that the discussion focused more on comparison and analysis with existing literature.

Through these adjustments, we hope that the discussion section can more effectively showcase the position of our research within the existing knowledge system, as well as the contribution of our results to related fields. Thank you again for your valuable feedback. We look forward to your further feedback.

Comment 4: The conclusion remains inadequate and requires rewriting.

Response:

Thank you to the reviewer for reviewing and providing feedback on our work. Based on your suggestion, we have rewritten the conclusion section. The new conclusion provides a clearer summary of the main findings of the study and further emphasizes the contribution and significance of these findings to related fields.

We believe that these modifications make the conclusion section more comprehensive and in-depth, better aligning with the overall goals and outcomes of the research.

Thank you again for your valuable feedback. We look forward to your further guidance.
