# Supplementary material for: Long-term consumption of hydrogen-rich water provides hepatoprotection by improving mitochondrial biology and quality control in chronically stressed mice
Source: PLoS One. 2025 Feb 14;20(2):e0317080. doi: 10.1371/journal.pone.0317080 (PMC11828380; doi:10.1371/journal.pone.0317080)
Supplement: Attachment — Submitted filename: Response to Reviewers.docx [file pone.0317080.s006.docx]

Reviewer #1:

Comment 1：In the result part, the figures names should not be written in the text. Each figure should be followed by its name.

Response: Thank you for your thorough review and valuable feedback on my manuscript. Regarding your comment about “the figure names should not be written in the text; each figure should be followed by its name,” I will carefully consider this and make the necessary revisions. In the revised manuscript, I will ensure that figure names are not mentioned in the text and that each figure is followed by its corresponding name. Thank you again for your constructive suggestions; I will work to enhance the quality of the manuscript.

Comment 2&3: While the manuscript was significantly improved and it present a solid foundation, the discussion part it is not strongly made in its current form.
The discussion part lacks the depth, critical analysis, and clinical relevance needed (Strong discussions connect animal model findings to potential human applications, or at least acknowledge the challenges of translation) to make it truly strong. Addressing these aspects would significantly enhance the quality of this Manuscript.

Response：Thank you for your thorough review and constructive feedback on my manuscript. I greatly appreciate your comments regarding the discussion section. I recognize that the discussion lacks depth, critical analysis, and clinical relevance, and I will take this feedback seriously as I work on the revisions.
In the revised manuscript, I will enhance the discussion section, particularly by making stronger connections between findings from animal models and potential human applications, as well as addressing the challenges associated with translation. I believe these modifications will significantly improve the overall quality of the manuscript.
Thank you once again for your valuable insights; I will strive to make the manuscript more robust and clinically relevant.
